# Hypoglycemia with lactic acidosis caused by a new MRPS2 gene mutation in a Chinese girl: a case report

**DOI:** 10.1186/s12902-021-00924-1

**Published:** 2022-01-06

**Authors:** ChangZhi Liu, WeiRan Zhou, QuanE Liu, ZaiXin Peng

**Affiliations:** 1 Xiangxi Tujia and Miao Autonomous Prefecture People’s Hospital, Jishou, China; 2 Jinan Children’s Hospital (Qilu Children’s Hospital of Shandong University), Jinan, China

**Keywords:** *MRPS2*, Hypoglycemia, Lactic acidosis, Case report

## Abstract

**Background:**

Mitochondrial ribosomal protein S2 (*MRPS2*) gene mutation, which is related to severe hypoglycemia and lactic acidosis, is rarely reported globally.

**Case presentation:**

We report a case of a new *MRPS2* gene mutation in a Chinese girl who presented with hypoglycemia and lactic acidosis. A homozygous C.412C > G variant that could cause complex oxidative phosphorylation deficiency and had not been reported before was identified. The clinical manifestations included recurrent vomiting, hypoglycemia, lactic acidosis, sensorineural hearing loss, and gall bladder calculi. Hypoglycemia and lactic acidosis improved after the administration of sugary liquid and supportive treatments.

**Conclusions:**

Recurrent hypoglycemia with lactic acidosis and sensorineural hearing loss should lead to suspicion of mitochondrial defects and the early refinement of genetic tests.

## Background

Hypoglycemia with lactic acidosis is rarely seen in clinical practice. Despite early treatment, hypoglycemia with lactic acidosis still has a high mortality rate. The main causes of hypoglycemia and lactic acidosis include the blood system of malignant tumor, septicemia, renal insufficiency, malaria, certain drugs, and mitochondrial dysfunction [1].

Mitochondrial diseases are important causes of hypoglycemia and lactic acidosis. Defects in mitochondrial translation in mitochondrial diseases could lead to complex oxidative phosphorylation system (OXPHOS) deficiency, which may cause severe multisystem dysfunction early in life and even death [2–4].

Most of the mitochondrially encoded proteins are core subunits of OXPHOS comprising the respiratory chain and ATP synthase [5]. The biogenesis of mitochondrial OXPHOS depends on mitochondrial-specific ribosomes in the mitochondrial matrix for the translation of 13 mtDNA-encoded polypeptides [4]. The mitochondrial genetic system takes charge of the post-translation maturation of the newly synthesized polypeptides. However, how these activities are organized and coordinated is largely unknown [5].

Mutations in nine mitochondrial ribosomal protein-encoding genes, including mitochondrial ribosomal protein S2 (*MRPS2*), have been reported. *MRPS2* mutations have been found in two patients [4]. In the present study, we report a case of recurrent hypoglycemia with lactic acidosis in a school-aged child caused by a new *MRPS2* mutation, which has not been reported, to reveal a rare cause of hypoglycemia and lactic acidosis in *MRPS2* mutation and learn the phenotype of the *MRPS2* gene.

## Case presentation

The girl aged 6.5 years old was hospitalized because of recurrent vomiting for more than 2 years. She had poor appetite and hypoglycemia during hospitalization but without seizures and unconsciousness. Her condition improved after the administration of sugary liquid and supportive treatments. However, her hypoglycemia was recurring. The girl was taciturn with language retardation. She could only say simple words and sentences, but her motor development was normal. When she was 4 years old, she had sensorineural hearing loss and was treated by parallel cochlear implants. When she was 4 years and 9 months old, she had gall bladder calculi due to chronic cholecystitis, which was accompanied by erosion and bile reflux, and was treated by laparoscopic cholecystectomy under general anesthesia. She was G2P2 and born naturally at term. Her brother was healthy, and the siblings had no similar medical history or family history.

Physical examinations revealed no abnormal findings. Muscle tension and tone were normal. The results of blood tandem mass spectrometry and urine gas chromatography were normal. Blood glucose was 1.2–2.5 mmol/L when she was hospitalized. She had metabolic acidosis (pH: 7.224 [reference value: 7.35–7.45], HCO3−: 4.3 mmol/L [reference value: 22–26 mmo/L), base excess: − 24 mmol/L (reference value: 3 mmol/L), lactate: 8.9 mmol/L [reference value: 0.5–1.5 mmol/L]) and normal liver enzyme, blood lipid, myocardial enzyme level, and thyroid function.

**Fig. 1:**
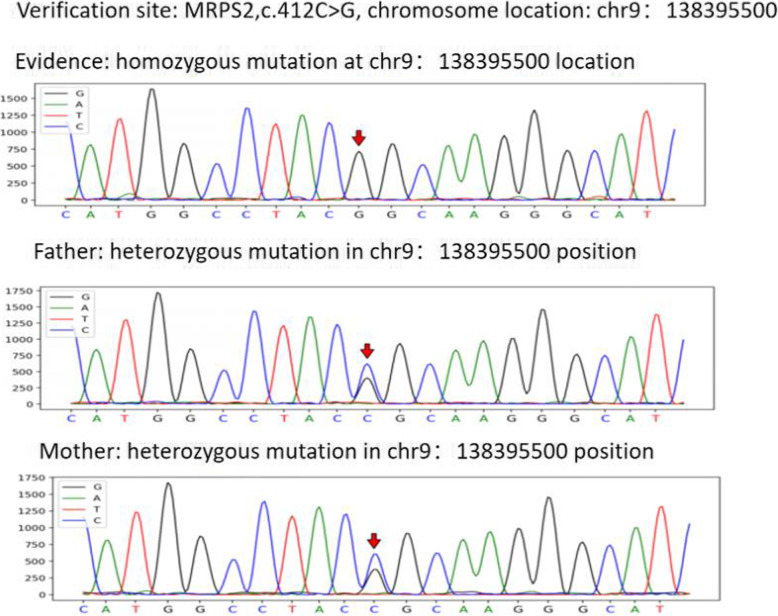
Genomic sequences of the *MRSP2* gene. The proband had the homozygous mutation C. 412C>G(p.R138G). The parents of the proband were all heterozygous for this mutation

The cause of the recurrent hypoglycemia was unknown; therefore, a gene test was performed, and a new homozygous *MRPS2* gene mutation, C.412C > G (p.R138G), was found (chromosome location: chr9:138395500), as shown in Fig. 1.

## Treatment and prognosis

The child was repeatedly hospitalized because of vomiting and hypoglycemia. She was treated by glycemia. Her appetite recovered as her blood glucose became normal. Her hearing loss was corrected and her speech development improved after cochlear implantation and speech rehabilitation, respectively. The current course of the disease was more than 2 years. The girl was 7 years old with a weight of 15.6 kg and a height of 111 cm. Her language developed normally. She was rated as moderate by Baby–Junior Middle School Students Social Living Ability Scale, and she could attend kindergarten like normal children.

## Discussion and conclusions

This case is the first Chinese case of *MRPS2* gene mutation, which has the potential to lead to OXPHOS deficiency. Gardeitchik et al. [4] reported two children with *MRSP2* mutation for the first time in 2018 with clinical manifestations of sensorineural deafness, hypoglycemia, lactic acidosis, 2-oxoglutarate acidurea, and developmental retardation. Both children survived and were 11 years old during the follow-up. They used fibroblasts obtained from skin biopsies of both subjects to characterize the effects of the identified variants and found that *MRPS2* mutation makes the protein unstable and thus damages the assembly of the small subunit mitochondrial DNA (MT-SSU), a part of a dedicated translation machinery that could execute mitochondrial translation. In other words, the reduction of MT-SSU assembly caused by *MRPS2* mutations could lead to the inhibition of mitochondrial translation and a variety of OXPHOS defects.

*MRPS2* was also studied in tumors. *MRPS2* upregulated in patients with follicular thyroid tumors caused by dysfunctional mitochondrial metabolism [6]. The ribosomal fraction in patients with mutations of mitochondrial small subunit ribosomal proteins, *MRPS16* and *MRPS22*, is still about 60% of the control levels of *MRPS2*. This result suggests that the stability of *MRPS2* is not strongly affected. Indeed, *MRPS2* may play a major structural role [7].

In this case, a new mutation, C.412C > G, was found in *MRPS2*. The homozygous mutation is consistent with the autosomal recessive genetic pattern of the disease, and the gene-associated disease is consistent with the clinical manifestation of the proband. Notations according to the American College of Medical Genetics and Genomics Standards and Guidelines are as follows: PM1: the variation is located in a functional domain; PM2: the variation is absent from controls in the Exome Sequencing Project, 1000 Genomes Project, or Genome Aggregation Database; PP3: Sift, PolyPhen-2, and MutationTaster show multiple lines of computational evidence to support a deleterious effect on the gene or gene product. Therefore, the variation was considered pathogenic. Regretfully, we did not perform further experiments to prove the functions of the variant.

We paid attention to other *MRPS* mutations. Ninety-eight mitochondrial protein-coding genes have been reported to date. Among the patients, two siblings had *MRPS7* mutation (MIM: 611974) [8]; one had *MRPS16* mutation (MIM: 609204) [9]; five had *MRPS22* mutation (MIM: 605810), including 3 siblings [10]; and the rest had mutations in *MRPS34* (MIM: 611985) [11], *MRPL3* (MIM: 607118) [12], *MRPL12* (MIM: 602375) [13], *MRPL44* (MIM: 611849, [14], and *MRPS2* [4]. A total of 24 subjects have been reported, all of whom had disease onset as newborn or infants. Defects in *MRPS22* and *MRPS34* led to early fatal phenotypes. One case with *MRPS16* defect died 3 days after birth; four of the five cases with *MRPS22* defects died early, and two of the cases with *MRPS34* defects died in infancy because of respiratory failure. In addition, two of the four siblings with defects in *MRPL3* died at 15 and 17 months, whereas the other two siblings were still alive at 3 years of age. Among the cases with *MRPS7* defects, one case died at 14 years old, and the others were followed up to 1.5–26 years old. The rest were survival cases, and the oldest one had a *MRPL44* defect.

Clinically, two patients with *MRPS23* [15] defect reported hypoglycemia. In addition to *MRPS23*, all patients had lactic acidosis. The remaining clinical manifestations included cardiac involvement, such as cardiomyopathy, retinitis, redundancy of the skin of the neck, cranial deformity, and hearing impairment.

This case is the first reported *MRPS2* mutation among Chinese. The girl suffered from recurrent hypoglycemia, lactic acidosis, and sensory neurologic deafness with normal intellectual and motor development. The mechanism of the new *MRSP2* mutation needs to be further studied, and the prognosis still needs to be confirmed by long-term follow-up. When we encounter school-aged children with hypoglycemia accompanied by lactic acidosis, we should pay attention to mitochondrial diseases after excluding infection, malignant tumor, drugs, and other factors. Mitochondrial gene detection can help detect related mutations and make an accurate diagnosis.

## Data Availability

The data that support the findings of this study are available from the. corresponding author upon reasonable request.

## Abbreviations

*MRPS2*: Mitochondrial ribosomal protein S2
OXPHOS: oxidative phosphorylation system
